# Long-acting parenteral formulations of hydrophilic drugs, proteins, and peptide therapeutics: mechanisms, challenges, and therapeutic benefits with a focus on technologies

**DOI:** 10.1007/s13346-024-01747-y

**Published:** 2024-12-11

**Authors:** Deepa D. Nakmode, Baljinder Singh, Sadikalmahdi Abdella, Yunmei Song, Sanjay Garg

**Affiliations:** Centre for Pharmaceutical Innovation, University of South Australia, North Terrace, Adelaide, SA 5000 Australia

**Keywords:** Small molecules, Hydrophilic, Long-acting, Sustained release, Peptides, Injectable

## Abstract

Despite being the most widely prescribed formulation, oral formulations possess several limitations such as low adherence, low bioavailability, high toxicity (in the case of anticancer drugs), and multiple-time administration requirements. All these limitations can be overcome by long-acting injectables. Improved adherence, patient compliance, and reduced relapse have been observed with long-acting formulation which has increased the demand for long-acting injectables. Drugs or peptide molecules with oral bioavailability issues can be easily delivered by long-acting systems. This review comprehensively addresses the various technologies used to develop long-acting injections with a particular focus on hydrophilic drugs and large molecules as well as the factors affecting the choice of formulation strategy. This is the first review that discusses the possible technologies that can be used for developing long-acting formulations for hydrophilic molecules along with factors which will affect the choice of the technology. Furthermore, the mechanism of drug release as well as summaries of marketed formulations will be presented. This review also discusses the challenges associated with the manufacturing and scale-up of the long-acting injectables.

**Graphical abstract:**

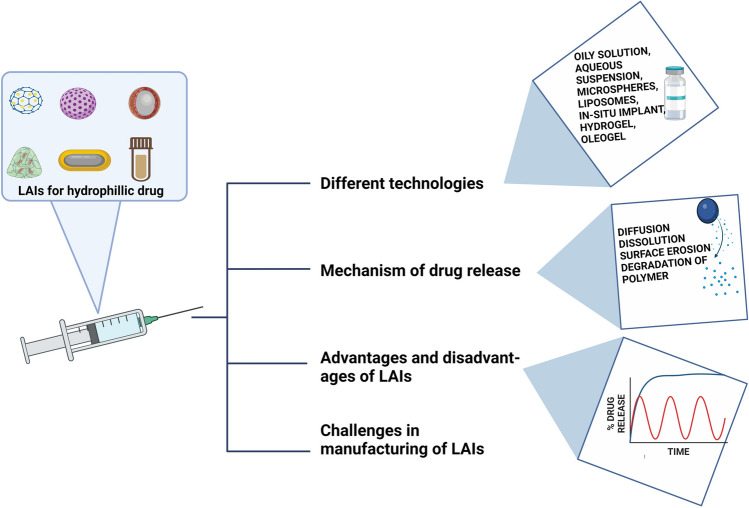

## Introduction

Long-acting injectables (LAIs) are the class of parenteral drug delivery systems designed to offer prolonged or sustained release of drug molecules over a prolonged period [1]. It presents several benefits over conventional formulations, including extended curative effects, reduced toxicity, reduced necessary dosage, enhanced adherence, and reduced dosing frequency. Additionally, it avoids hepatic first-pass metabolism and gastric degradation, making it a promising option for delivering drugs that undergo pre-systemic metabolism. As a result, LAIs attracted significant attention in the last decades. Of note, rapid growth has been observed for the injectables and delivery devices in the market. Even though the global consumption of solid oral products is more compared to that of injectables, there has been a noticeable increase in the delivery of drugs parenterally [2] Due to their susceptibility to degradation by enzymes in the GI tract, faster clearance, and the short half-life in the blood [3, 4], Peptides are generally potent with low daily requirements, hence formulating LAI is feasible.

LAI formulation was first introduced in the 1960s for the antipsychotic drug which was developed by E.R. Squibb and Son [5] as an oil-based system limited to lipophilic or hydrophobic molecules. Over time different strategies evolved for accommodating different drug molecules. For delivering hydrophilic molecules specifically peptides and proteins, microsphere systems were introduced in the 1990s. Besides microsphere systems were protected by patents, which led to the introduction of alternative systems such as in-situ forming implants from 1998 to 2005 [6].

**Fig. 1:**
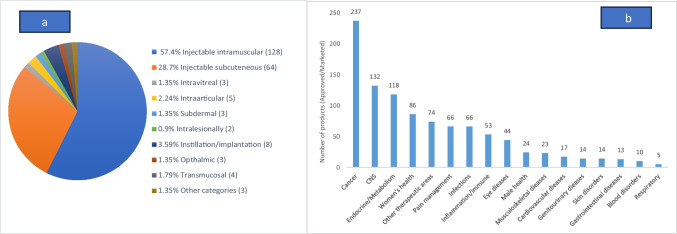
Current landscape of approved LAIs (**a** and **b**) figure reproduced, with permission, from [7]

There are a total of 223 marketed long-acting formulations [7]. Figure 1a illustrates the classification of approved LAIs based on the route of administration and 1b therapeutic areas. The current trend shows an increase in chronic disease numbers that require treatment for the long term, including cancer [8], neurological diseases [9], metabolic diseases [10], cardiovascular diseases [11], and mental disorders [12]. Most of these diseases require a constant plasma level of the drug to show prolonged efficacy while reducing the unwanted side effects [13]. As shown in Fig. 1b highest number of LAIs approved are for cancer treatment followed by central nervous system therapy.

Molecules administered by conventional parenteral formulations such as solutions and suspensions have a short life which increases the frequency of dosing and are cleared from the body, to resolve this issue LAI provide sustained release of the drug for a prolonged duration without variations in drugs plasma concentration [14, 15]. In addition to this, LAI systems have the potential to extend the systemic or local exposure of the drug for a prolonged period after a single administration [16, 17]. The advantages and disadvantages of the long-acting delivery systems have been discussed briefly in Table 1. Over the last few years, a significant increase in the development of LAIs, with different strategies, has been observed. Hydrophobic drugs are often easily prepared into aqueous or oily suspension-based LAI for the subcutaneous (SC) or intramuscular (IM) route. Nevertheless, a large number of parenterally administered drugs are water soluble, and controlling the release of such class drugs requires the use of less water-soluble polymer [14].

**Table 1:** Advantages and Limitations of the long-acting injectable

Advantages	Limitations
**Reduced Dosing Frequency:** LAIs decrease the need for frequent dosing compared to oral administration	**Limited Dose Adjustments:** It is challenging to alter the dosage after administration of LAIs
**Enhanced Patient Compliance:** LAIs contribute to improved patient adherence, addressing both intentional and unintentional nonadherence to therapy	**Injection Site Pain:** Patients may experience pain at the injection site, which can be a drawback of this form of medication
**Decreased Relapse Cases:** LAIs result in fewer relapse instances, thereby alleviating the burden on the healthcare system	**Perception of Control:** Some individuals may feel a sense of being controlled when receiving medication through injections, impacting their psychological well-being
**Improved Bioavailability and Pharmacokinetics:** LAIs enhance the bioavailability and pharmacokinetic profiles of drugs	**Risk of Dose Dumping:** LAIs pose a risk of dose dumping, where a large amount of medication is released rapidly, potentially leading to adverse effects. Where removal of the implant and antidote may be required to manage the side effects
**First-Pass Metabolism Bypass:** The administered dose is not affected by first-pass metabolism, leading to increased therapeutic output	**Higher Comparative Cost:** LAIs are relatively more expensive compared to alternative forms of medication administration
**Enhanced Tolerability:** LAIs generally exhibit improved tolerability with fewer side effects observed in many cases	**Challenges in Application:** The process of administering the treatment may be inconvenient, posing difficulties or hindrances in the application
**Effective for Chronic Conditions:** LAIs are a preferable route of administration over oral methods for treating chronic conditions	**Challenges of irreversible therapy:** Inability to discontinue therapy once initiated poses risks of severe side effects and toxicities
**Reduced Monitoring Frequency:** Less frequent monitoring is required with the use of LAIs	The requirement of highly potent drugs to control the dose volume
**Prevention of Drug Abuse:** LAIs contribute to preventing drug abuse due to their specific formulation and administration	Other limitations like PK tail and emergence of resistance due to continuous exposure can be seen

Depending on the physiochemical properties of active pharmaceutical ingredients (API) (such as hydrophobicity, stability, log P, and water solubility), disease condition, duration of treatment, and patient condition the strategy for designing a control release system will vary [18]. Of note modulating the release of the hydrophilic drugs is difficult due to their high solubility.

In this review, we aim to discuss the possible technologies that have been explored for the development of LAIs containing hydrophilic small molecules or proteins along with the examples of the approved formulation. Lastly, the release mechanism from different systems have also been discussed in brief.

## Drug delivery technologies for LAIs

**Fig. 2:**
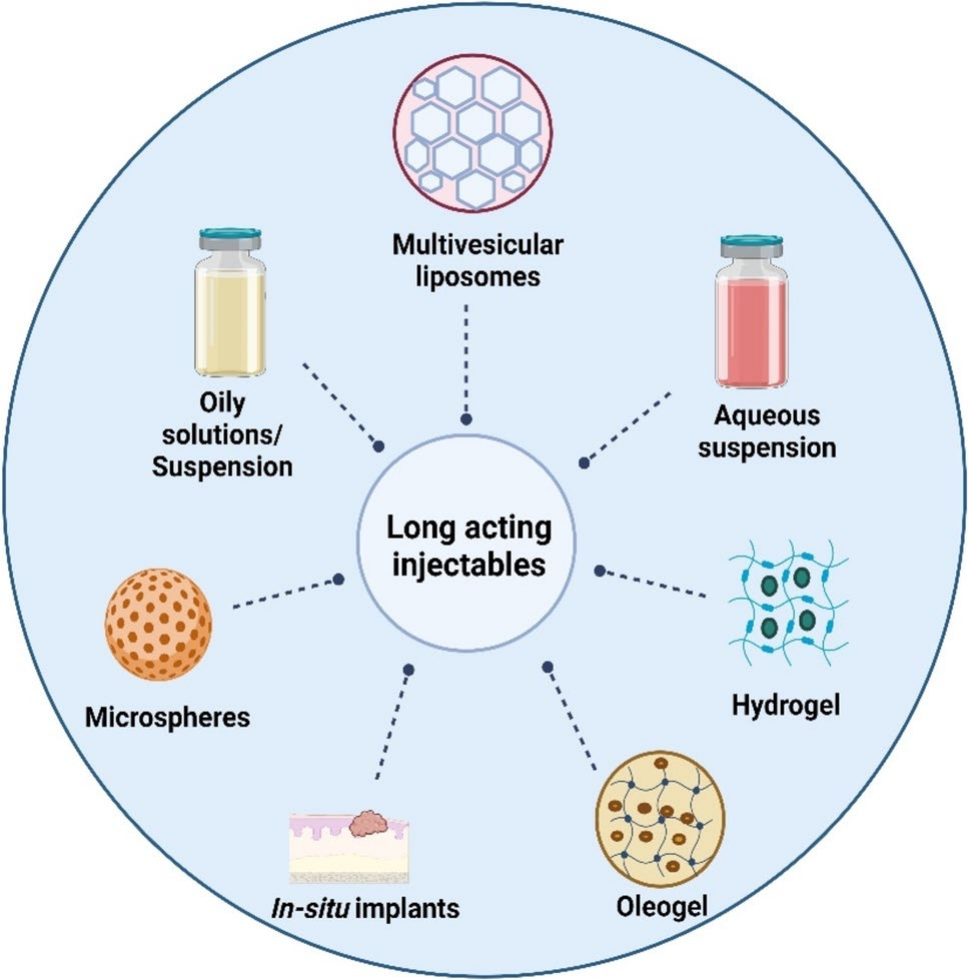
Long-acting formulation strategies for hydrophilic molecules (created with biorender)

Various approaches have been successfully used for preparing long-acting injectables for hydrophilic drugs such as the conversion of water-soluble API into lipophilic pro-drug, polymeric microsphere, hydrogel, in-situ depot, oleogel preparation multivesicular liposomes, implantable systems, long-acting microneedle (illustrated in Fig. 2).

### Prodrugs

Several long-acting injectables have been approved by tailoring the drug molecules at molecular as well as particulate levels, Covalent linking of fatty acyl chain to the molecules to form ester pro-drug, which increases the lipophilicity and enhances partitioning in the fatty tissues has been the commonly used method [19]. The obtained lipophilic moiety is subsequently suspended or dissolved in oil to attain slow release of the drug over a prolonged period [20]. While developing oil-based systems viscosity of oil plays an important role, highly viscous oils are difficult to inject, whereas very low viscosity can release drugs rapidly on injection [21]. The pro-drug partition between the oil and the water phase can be changed by alteration of the fatty acyl chain length [19]. Although long-acting oily solutions and suspensions offer easy manufacturing and are cost-effective, it's applicable for potent drugs only due to the low volume and low drug loading offered by these systems [5]. Prodrug preparation using ethanoate, decanoate, and caproate has been successfully translated from the preclinical stage to the clinical stage [16]. Several oil-based long-acting formulations have been marketed (Table 2). Vegetable oils such as coconut oil, peanut oil, coconut oil, soyabean oil, safflower oil, sesame oil, and cottonseed oil are commonly used for the development of oil depot systems [22]. Nevertheless, the use of vegetable oils in the formulation has been associated with physical and chemical instability due to the presence of unsaturated fatty acids [23]. Synthetic oils such as such as ethyl oleate, medium-chain triglyceride, and isopropyl myristate can overcome stability issues.

**Table 2:** List of approved prodrug-containing oil-based systems along with the vehicle used for delivering those drugs

Brand name	Company	API	Dose strength	Clinical indication	Frequency of dosing (route, volume)	Oil vehicle
Androcur Depot	Bayer	Cyproterone acetate	100 mg/ml	Cancer	Every 2 weeks, IM (3 ml)	Castor oil
Clopixol Depot	Lundbeck Ltd	Zuclopenthixol decanoate	200 mg/ml	Schizophrenia	Every 4 weeks, IM (1–2 ml)	ViscoleoR a medium chain triglycerides/miglyols
Fluanxol Depot	Lundbeck Ltd	Fluphenazine decanoate	20–40 mg/ml	Psychotic disorders	Every 2–4 weeks, IM (0.25–2 ml)	Medium-chain triglycerides
Lyogen Depot	Lundbeck Ltd	Fluphenazine decanoate	7.5 mg 1-M, 22.3 mg 3-M, 30 mg 4-M, 45 mg 6-M	Schizophrenia	Every 4 weeks, IM (0.25–2 ml)	Sesame oil
Makena*	Lumara Health	Hydroxyprogesterone caproate	250 mg/ml	Preterm Birth	Weekly, IM (1 ml)	Castor oil, Benzyl benzoate
Naldebain ER	Lumosa Therapeutics Co., Ltd	Dinalbuphine sebacate	10 mg/ml, 20 mg/ml	Pain management	Weekly, IM	Sesame oil
Depixol	Lundbeck Ltd	Flupentixol decanoate	20 mg/ml	Antipsychotic	IM (2–3 ml)	Coconut oil

*—Withdrawn from the market because of lack of benefit observed for pregnant women [12]

Gaekens et al. prepared a depot formulation by synthesizing prodrug derivatives of nalmefene using palmitate (C16), octanoate (C8), decanoate (C10), and dodecanoate (C12) in sesame oil, which is used for treating alcohol disorder. The effect of the fatty chain length of lipids on the pharmacokinetic profile of dogs and minipigs after intramuscular injection at a dose of 5 mg-eq of nalmefene was evaluated. The results show a decline in C_max_ and an increase in T_max_ with an increase in the length of the fatty chain found. Prodrugs prepared with palmitate and dodecanoate maintained a therapeutic level of the drug for a 4-week period [24].

### Multivesicular liposomes

Multivesicular liposomes (MVLs) were initially described by Kim et al*.,* as lipid bilayer vesicles arranged in concentric circles or non-concentric conjoined vesicles [25]. Based on the nature of the drug molecules, the hydrophilic drugs are entrapped in an inner layer which is aqueous, and lipophilic drugs are in a lipid bilayer [26]. The average diameter of MVLs ranges from 1–100 µm [27] where each aqueous polyhedral compartment is separated by a lipid bilayer membrane [28]. A commonly used technique for preparing MVLs is a double emulsion, a drug containing lipid vesicles formed by the first emulsion by adding the drug into the water phase, and then in the second emulsion lipid vesicles join to form a conjoined structure which on solvent removal forms MVLs. The solubility of API in the initial solution must be high enough to prevent precipitation in the second emulsion to attain the required drug loading. Therefore, drug molecules with high water solubility or ionizable drugs that show pH-dependent solubility are selected for MVLs. MLVs are preferred for hydrophilic drugs due to their high-water content (95%). The depot effect of this system depends on the reduced clearance, the stability of liposomes is increased due to the non-concentric layer which in addition reduces the burst effect and prolongs the release of the drug [29]. Despite its clinical success MVLs preparation has several drawbacks due to its vast characterisation and the manufacturing process necessitates an aseptic environment throughout the process [30]. The complex structure and size, along with the distinctive release pattern resulted in a complex pattern of release [31, 32].

Formulation components of MVLs mainly include cholesterol for maintaining the lipid bilayer stability, phospholipids which form the bilayer, and branched non-polar lipid which acts as bridging agents within the vesicles [26]. Table 3 is the summary of approved MVLs formulations along with their formulation composition. MLVs have also been used for encapsulation of other hydrophilic molecules such as ropivacaine hydrochloride [33], acyclovir sodium [34], interferon alpha-2b [35]and bupivacaine [36]. Shen et al*.* developed an MVL formulation for ropivacaine hydrochloride and evaluated its in-vivo sustained release. Multiple emulsion technique was used for preparing MVLs. The first W/O emulsion was prepared using egg phospholipid, cholesterol, and triolein. In the second step, the water phase with L-lysine and glucose was added to the first emulsion resulting in the precipitation of MVLs. The characterization of the prepared MVLs was carried out such as determination of particle size, zeta potential, encapsulation efficiency (EE), and the in-vitro drug release studies. In-vitro drug release followed first-order kinetics with slow drug release up to 48 h, in-vivo pharmacokinetics showed prolonged T_1/2_ of the drug with a decrease in C_max_ which signifies reduced toxic effects of the drug [33].

**Table 3:** Multivesicular liposomal formulations encapsulating hydrophilic drug approved by FDA

Brand name	Manufacturer	Drug	Water solubility	Dose strength	Clinical indication	Dosing frequency, route (volume)	Composition
Depocyt	Pacira Pharmaceuticals	Cytarabine	2.8 mg/ml	10 mg/ml	Lymphomato-us meningitis	Intrathecal (5 ml)	cholesterol, triolein, dioleoyl phosphatidylcholine (DOPC), and dipalmitoylphosphatidylglycerol (DPPG)
DepoDur	SkyePharma Inc	Morphine sulfate	10 mg/ml	10 mg/ml	Post-surgical pain relief	Epidural injection (1 ml)	1,2-dioleoyl-sn-glycero-3-phosphocholine (DOPC), cholesterol, 1,2-dipalmitoyl-sn-glycero-3-phospho-rac-(1-glycerol) (DPPG), tricaprylin and triolein, 0.1 mg/mL
Exparel	Pacira Pharmaceuticals	Bupivacaine	0.097 mg/ml	13.3 mg/ml	Post-surgical pain relief	Infiltration	Dierucoylphosphatidylcholine (DEPC), Dipalmitoylphosphatidylglycerol (DPPG), Cholesterol for parenteral use, Tricaprylin

MVLs have also been used for the encapsulation of hydrophilic large molecules such as interferon α-2b, Qiu et al., prepared MVLs loaded with interferon α-2b for obtaining the prolonged release of interferon. The prepared MVLs was tested in the healthy mice after SC administration, where the concentration of interferon in blood was evaluated by ELISA method (enzyme-linked immunosorbent assay). The double emulsion technique was utilized for preparing the MVLs, which were further evaluated for EE, size distribution, and in-vitro drug release followed by an in-vivo PK study. A significant amount of interferon was detected in the serum for 5 days with an estimated half-life of 30 h. Along with the ability to provide sustained release, MVLs also provide the advantage of maintaining the structural stability of the protein [35].

A short half-life drug has also been encapsulated into MVLs for prolonging the concentration of the active moiety in the body. In the experiemnt performed by Abuzar and the team, they prepared the intraperitoneal oxaliplatin encapsulated MVLs for the treatment of colorectal cancer. The MVLs were prepared by double emulsion technique which showed good EE i.e. 92.16% ± 2.17%. In-vitro drug release demonstrated burst release followed by lag phase with drug release up to 6 days. In-vivo pharmacokinetic studies after IP administration of formulation showed improvement in the bioavailability with longer mean residence time with the release of oxaliplatin slowly due to slow erosion of MVLs [37].

MVLs provide several advantages over multilamellar liposomes such as higher encapsulation efficiency and prolonged drug release. Jain et al. developed the MVLs of acyclovir sodium and compared the release and pharmacokinetics with multilamellar liposomes. Acyclovir-loaded MVLs were made by the reverse phase evaporation method. The formulated liposomes were evaluated for size distribution, EE, and in-vitro drug release. The loading capacity of the prepared liposomes was 3 to 6 times higher than the multilamellar liposomes, in addition to this, the in-vitro release profile of acyclovir from MVLs shows up to 70% release in 96 h whereas the multilamellar liposomes 80% drug got released from in 16 h. The AUC _0–48_ of acyclovir from MVLs was 1.5 folds higher than the one from conventional liposomes which proves that the MVL provides high loading and more sustain release of hydrophilic drug [34].

Vatankhah and the team explored the application of MVL for loco regional drug delivery for delivery of vancomycin for treating osteomyelitis. Vancomycin was loaded in empty MVLs by the active loading method. In-vitro release results demonstrated sustain release of vancomycin for up to 19 days from the optimized formulation. In-vitro assay for testing anti-bacterial activity showed effective anti-bacterial activity against the osteomyelitis-causing bacteria [38].

### Polymeric microspheres

Polymeric microspheres have been effectively utilized for encapsulating small drug molecules and proteins [39]. Microsphere formulation involves encapsulation or entrapment of hydrophilic to lipophilic drug molecules. Generally, polymers employed in microsphere preparation are biocompatible and biodegradable [1]. Microspheres with the size ranging from 10–250 µm are desired due to their easy injectability with standard needles, in addition to preventing the local tissue inflammation and reducing phagocytosis [40–42], Microspheres prepared by traditional techniques such as solvent evaporation/ extraction and coacervation show uneven particle size distribution with inadequate injectability of the formulation [43], this limitation has been overcome by novel techniques such as membrane emulsification and microfluidics [44]. Various polymers are used in the preparation of microspheres including natural polymers example proteins (gelatine [45], albumin [46], polysaccharides (chitosan [47], dextran), and synthetic polymers for example poly (lactic acid), poly (glycolic acid), a copolymer of polylactic and polyglycolic acid (PLGA) [48]. The advantage of the use of PLGA in the microsphere is drug release modification can be done by changing the drug-to-polymer ratio or changing the molecular weight of the polymer or the ratio of glycolic to lactic acid [49, 50]. Microsphere formulation has been widely used for delivering the peptide and protein molecules, thereby preventing degradation by enzymes, and providing sustained release for a longer time [51]. Most of the approved marketed microsphere formulations include encapsulated protein or peptide molecules (Refer to Table 4). The limited loading capacity of up to 25% w/w is the major drawback of the microsphere systems, which makes it difficult to load the low potency drugs due to high dose requirement. It directly affects the injectability, generally suspension of more than 40% w/v of microsphere results in high viscosity [16].

**Table 4:** List of marketed microsphere-based long-acting injectables

Brand name	Manufacturer	Active moiety	Clinical indication	Dosing frequency, route (volume)	Encapsulation technique used	Polymer used
Bydureon^®^ 2 mg	AstraZeneca	Exenatide	Type 2 diabetes mellitus	Weekly, SC	Coacervation	PLGA_75:25_
Lupron Depot^®^ 7.5 mg- 1 M, 22.5 mg- 3 M, 30 mg- 4 M, 45 mg- 6 M	Takeda, Abbott	Leuprolide Acetate	Breast and prostatic cancer	4–26 weeks, IM	Emulsion solvent evaporation	PLGA_75:25_
Nutropin depot* 13.5 mg, 18 mg, 22.5 mg	Genentech Inc	Somatotropin	GH deficiency	4–5 weeks, SC	Emulsion method	PLGA_50:50_
Suprecur MP	Sanofi	Buserelin acetate	Endometriosis	Monthly, SC	NA	PLGA
Signifor 20 mg	Novartis	Pasireotide pamoate	Acromegaly	Every 4 weeks, IM	Emulsion solvent evaporation	PLGA_50:50_
Sandostatin LAR depot 20 mg	Novartis	Octreotide acetate	Carcinoid tumors, acromegaly	Monthly	Coacervation	PLGA glucose star polymer
Somatuline Depot 60 mg	Ipsen	Lanreotide acetate	Acromegaly	4 weeks	Spray drying	PLGA

*—withdrawn from the market due to scale-up issues [61]

Amongst the different available techniques, solvent evaporation is extensively utilized for preparing microspheres. The solvent evaporation technique involves, numerous emulsification methods are there such as oil in water (O/W), water in oil in water (W/O/W), and solid in oil in water (S/O/W), the selection of the emulsion method is solely dependent on the aqueous solubility of drug molecule or peptide [52, 53]. Based on the manufacturing process both hydrophilic and lipophilic drugs can be encapsulated in microspheres [53].

Zhang et al. prepared a microsphere encapsulating peptide analogue of the luteinizing hormone-releasing hormone (LHRH), triptorelin acetate using PLGA _75:25_ by W/O/W emulsion method. PLGA at 15% concentration with 1% polyvinyl alcohol and 0.1 M sodium acetate as an aqueous phase gave optimized microspheres with an encapsulation efficiency of 63.5% ± 3.4% and 35.3 ± 1.8 µm average volumetric particle size. The formulations demonstrated a multiphasic release profile with optimized formulation showing sustained release of triptorelin acetate over 63 days, which demonstrates that microsphere technology can be used for peptide molecules as well [54].

New technology such as microfluidics has also been explored in the formulation of microspheres by Choi et al., where they prepared donepezil microspheres using PLGA of different grades. All the prepared microspheres were characterized for EE, surface morphology, particle size distribution, and in-vivo pharmacokinetics in beagle dogs. The optimal formulation was prepared using PLGA _75:25A_ at 15% concentration giving a microsphere with a median diameter of 40.84 µg and encapsulation efficiency of 84.9%. In the in-vivo PK study, the formulation demonstrated the least initial burst release, with Cmax = 3.68 µg/ml-maintained plasma concentration in dogs for a month [55].

Hydrophilic drugs possess low EE when formulated in microsphere formulation by the traditional method, to overcome this limitation novel method was utilized known as post-loading mode by Wen et al., where porous PLGA microspheres were prepared first followed by loading of ropivacaine hydrochloride a hydrophilic drug by suspending blank microspheres into saturated ropivacaine hydrochloride solution where drug slowly diffused into the porous microspheres. Drug loading by this method was found to be 8.72%. The efficacy of the microspheres was evaluated in a rat sciatic nerve block model where prepared microspheres were compared to the FDA-approved bupivacaine liposomal suspension. Prolongation in the nerve block time was observed in the case of prepared microspheres in addition the duration of analgesia was 5Vh, which was 1.7 folds longer than the bupivacaine formulation [56].

In addition to the low EE, microspheres also show a very high initial burst release of the drug. The study done by Liu et al. involved hydrophobic ion-pairing complex formation for octreotide acetate. Dextran sulfate sodium was selected for hydrophobic ion pairing because of its high efficiency in binding to the drug along with dissociation ability when the counter ions are present. The prepared complex was encapsulated into the microsphere by the specific solid in oil in water (S/O/W) emulsion technique. The pores were healed at the temperature of 40 °C for the 6-h time, which was confirmed by scanning electron and fluorescence microscopy. The EE of the microspheres increased from 44 to 90% by this strategy. The in-vitro release profile showed sustained drug release up to 35 days with the reduced burst release from 16.81% to 3.56% for S/O/W emulsion-prepared microspheres. In in-vivo experiments, steady drug release was observed along with a stable blood level of octreotide acetate which was confirmed with ELISA kit [57].

Microsphere systems have also been used successfully for intravitreal drug delivery of hydrophilic drugs for the treatment of glaucoma, the conventional eye drops are associated with low residence time and low bioavailability due to the poor permeability via cornea leading to multiple times application of eye drops affecting the adherence [58].

Mietzner et al., developed fasudil microspheres for intravitreal delivery as vitreous provides a huge space of about 100 µl as a drug reservoir. The hypothesis was the transport of fasudil ROCK inhibitor towards the anterior chamber from the depot without exposure to the ocular surface. The ester terminated PLGA 50;50 was used for the preparation of microspheres. A double emulsion solvent evaporation technique with some modification was used for preparing microspheres. When the concentration of the polymers were increased, the particle size of the particles were also increased. The smallest microspheres (3.4 µm) prepared with 25 mg/ml PLGA showed the highest burst release of up to 65% in 3 days followed by sustained release up to 20 days whereas medium microspheres prepared with 67 mg/ml PLGA showed up to 40% burst release followed by sustain drug release up to 35 days. When the drug was encapsulated as a solid with 200 mg/ml PLGA, a microsphere with size 66.9 µm was obtained, and a triphasic release profile was observed with 20% burst release with a lag phase up to 10 days followed by consistent drug release from 17 to 38 days. The biological activity of the prepared microspheres was tested by in-vitro cell-based assays using TM (trabecular meshwork cells) SC (Schlemm’s canal cells) and fibroblasts cells. The cytochemical staining confirmed the reduction in actin stress fibers in all three cell types [59].

Yang et al. encapsulated palonosetron hydrochloride by changing the pH of the external phase of the emulsion from 5 to 20 which showed the effect of pH on the encapsulation efficiency and the release profile. Microspheres were prepared by O/W and W/O/W methods, microspheres prepared by the O/W method with the external phase of pH 10 showed higher EE of 94.33% whereas by W/O/W emulsion method with up to 55% encapsulation efficiency observed. Surprisingly in the In-vitro release results the microspheres prepared with external phase pH lower than 7 showed lower initial burst release compared to the one prepared at a higher pH i.e., greater than pH 7. The microspheres prepared using pH 8 and 10 showed increased burst release by up to 28% could be due to the formation of pores. The microspheres (F7) with an EE of 86.51% and drug loading of 2.15% prepared with pH 7 external phase showed low burst release with zero order release kinetics were selected for pharmacokinetic studies. SC injection of F7 microspheres was given to the rats, and the delay in the elimination of palonosetron was observed with plasma concentration greater than 0.2 ng/ml up to 6 days [60].

### In-situ implants

In-situ implants are solutions or suspensions of drugs, in a polymeric solution prepared using an organic solvent [26]. Such systems are present in liquid or semisolid form, which upon injection spontaneously transforms into gel or solid depot at the site of injection [62]. Transformation into a depot follows different mechanisms such as in-situ precipitation*, *in-situ crosslinking, and in-situ gelling depending on the formulation composition. The implant formation due to in-situ precipitation occurs because of phase separation or solvent extraction or the conversion of solution to gel form due to pH [63] or temperature [62, 64]. Whereas implants formed due to in-situ gelling take place due to the cross-linking of monomeric or polymeric chains in response to ions [65] or enzymes or due to photoirradiation [66]. In-situ gelling occurs either because of the thermo-sensitivity of hot melts or the self-aggregation of liquid crystals in aqueous medium. The most researched approach for in-situ forming implants is in-situ precipitation with products successfully reaching the market [67]. This type of system consists of a biodegradable polymer such as PLGA, PLA, and water-miscible organic solvent e.g., NMP or DMSO with API dissolved or dispersed in the system [68]. The in-situ implant systems are excellent substituents for preformed implants and microsphere formulations [26], as they involve easy manufacturing and less equipment is required, in addition, they bypass the need for surgery to insert the implants [62]. Despite their easy manufacturing, these systems have an issue that needs to be resolved such as initial burst release, and irreproducible release profile due to variability in implant size and shape [62]. Table 5 compromises of list of FDA-approved in-situ forming implants delivering hydrophilic molecules.

**Table 5:** Examples of FDA-approved in-situ forming implants available in the market for hydrophilic APIs

Brand name	Manufacturer	Drug	Water solubility	Clinical indication	Dosing frequency, route (volume)	Polymer utilized
Atridox^**®**^ dental insert 450 mg	Atrix Laboratories Inc	Doxycycline Hyclate	50 mg/ml	Chronic adult periodontitis	Once every 4 months, Subgingival	PLLA dissolved in NMP
Eligard 30 mg	Tolmar Inc	Leuprolide acetate	250 mg/ml	Palliative treatment of advanced prostate cancer	Once every 4 Months, SC (0.5 ml)	PLGA Dissolved in NMP
Posimir 660 mg	Durect	Bupivacaine	0.097 mg/ml	Post-surgical anesthesia	5 ml	Sucrose acetate isobutyrate (SAIB)
SUSTOL 10 mg	Heron Therapeutics, Inc	Granisetron	0.434 mg/ml	Treatment of acute and delayed nausea and vomiting with chemotherapy	Weekly, SC (0.4 ml)	TEG-POE

Ahmed et al., Prepared an in-situ implant for montelukast sodium, a hydrophilic drug molecule utilized for treating asthma. The effect of PLGA _50:50_ was evaluated at different concentrations (10, 20, and 30%) in NMP and DMSO, ethyl acetate, and triacetin. The in-vitro release studies were carried at pH 7.4 in PBS (phosphate buffer saline) for all the formulations. The formulation containing 30% PLGA in NMP was selected as optimized due to its sustained release profile for 1 month with good syringeability. The in-vivo pharmacokinetics study was performed in rats for optimized formulation, in-situ implant for montelukast sodium demonstrated sustained release for the duration of 15 days following intramuscular injection [69].

Molinier et al. prepared hydrophobic salt of octreotide for improving the stability of octreotide in liquid formulations and evaluated the impact of salt formation on the stability and release behavior. Octreotide pamoate (Oct-pam) and sodium dodecyl sulfate salts (Oct-SDS) were prepared from octreotide acetate by counterion exchange technique. The prepared salts were dissolved into gel prepared by using triblock (Poly D, L lactic acid, and Polyethylene glycol) and diblock (methyl-PEG and poly lactic acid) by dissolving it in DMSO at varying concentrations. The in-vitro release studies were carried out at pH 7.4 by direct injection of formulation into release media, samples were withdrawn at different time intervals and analyzed by HPLC. The release studies showed reduced initial burst release and reduced release rate of octreotide from salt form compared to that of gel prepared with octreotide acetate (Oct-Ac). A similar pattern was observed in pharmacokinetics studies in rats. Reduced initial burst release was observed in Oct-pam and Oct-SDS formulation, with plasma concentration of octreotide calculated the C_max_ value for Oct-pam and Oct-SDS was reduced compared to Oct-Ac formulation [70].

Another approach involves microsphere incorporation in the in-situ gel prepared with sucrose isobutyrate (SAIB). Jiang et al., prepared the microspheres of rasagiline mesylate by emulsion solvent evaporation and phase separation method, which were further dispersed in the SAIB solution just before initiating in-vitro drug release studies and pharmacokinetic and pharmacodynamic studies. The microsphere with drug loading up to 30.12% and encapsulation efficiency of 89.88% were selected as the optimized microsphere. The in-situ gel formulations were tested for drug release, no burst release was observed in 24 h followed by prolonged release up to 60 days which confirms that the drug release can be controlled by dual controlled systems. The gel formulation containing 85:15 of SAIB and ethanol was selected for in-vivo study which was compared against the optimized microsphere formulation and the gel prepared with the drug. The peak plasma concentration of the drug after 1 h from the rasagiline microsphere loaded gel was 26.69 ± 8.56 ng/ml which was notably lower than the rasagiline microsphere 374.91 ± 121.28 ng/ml and rasagiline in-situ gel 274.93 ± 66.04 ng/ml after IM injection. Furthermore, the C_*max*_* /C*_*ss*_ of rasagiline microsphere loaded gel was 7.06 ± 1.16 ng/ml which was notably lower than the rasagiline microsphere 83.82 ± 27.06 ng/ml and rasagiline in-situ gel 79.75 ± 21.32 ng/ml which signifies the obvious reduction in burst release. The plasma concentration of rasagiline dropped below the quantification limit after 14 days for rasagiline microsphere and rasagiline in-situ gel, whereas rasagiline plasma concentration was still detectable after 32 days for rasagiline microsphere gel. This approach can be used for highly water-soluble hydrophilic small molecules [71].

In-situ implants provide an advantage for delivering peptides and protein molecules as it does not require any special equipment and tedious multiple steps for manufacturing. Liu and the team prepared a biodegradable in-situ implant of thymosin alpha. The thymosin alpha microparticles were prepared by freeze-drying thymosin alpha powder with chitosan as well as BSA. The freeze-dried powder was then suspended in the PLGA_50:50_ gel prepared by dissolving PLGA _50:50_ in NMP (N-methyl pyrrolidone) and triacetin. The in-vitro and in-vivo release studies indicated the sustained release for up to 4 weeks from in-situ forming implant prepared with thymosin chitosan microparticles. A good correlation was observed between in-vivo and in-vitro release with a linear correlation coefficient of 0.9899. The in-vitro cytotoxicity studies confirmed that the in-situ forming systems are safe for drug delivery [72].

### Long-acting hydrogel

Long-acting hydrogel systems are 3-dimensional networks formed by cross-linking of the polymer. The inner structure is usually a porous aqueous milieu which allows the loading of a various molecules such as genes, proteins, and small molecules [73]. Hydrogel systems transform from sol to gel on injection, due to external stimulation by pH, temperature enzyme, or chemical signal [74, 75]. Long-acting hydrogel systems possess several advantages such as biocompatibility, biodegradable, localized effect, and less drug interaction capability [13]. The hydrogel systems are administered by SC or IM route, acute inflammatory response is observed at the site of injection due to accumulation of macrophages and lymphocytes which disappears with time [76]. Despite several preclinical studies on hydrogels, there is no marketed long-acting hydrogel formulation yet [75–77].

Hydrogels that transform in response to temperature changes are called thermogel, the gelling temperature is mainly dependent on the chemical structure of the polymer, the hydrophobic chain length, and the concentration of the polymer [78]. The optimization of polymer concentration is essential to achieve satisfactory gelation temperature with acceptable viscosity for injection [79]. Thermo gel is most used due to ease of manufacturing which involves simple mixing of drug with polymeric solution at low temperature without loss of drug material, which is advantageous in the case of proteins and peptide molecules [73, 80, 81].

Li et al. explored the thermo-gelling property of synthesized triblock polymer of PLGA-PEG-PLGA in hydrogel to sustain the release of polypeptide i.e., exenatide. The in-vitro release profile exhibited initial burst release, which was reduced by the introduction of zinc acetate due to the formation of zinc-exenatide complexes in the gel matrix. The release of exenatide was consistently maintained for 7 days, whereas in-vivo pharmacokinetic study of the prepared hydrogel in mice model demonstrated better glucose tolerance up to 7 days on SC injection [82].

Thermosensitive gels provide an advantage of conversion from sol to gel at the injection site for localized drug delivery [83]. Pan and team attempted co-delivery of collagenase and trastuzumab-loaded thermosensitive hydrogel for peritumoral administration. PLGA-PEG-PLGA hydrogel was prepared by dissolving polymer (20%) in 0.9% NaCl solution at 4° C until a clear solution was obtained. Trastuzumab and collagenase were mixed with the blank hydrogel overnight until a clear solution was formed (Col/Tra/Gel) and (Tra/Gel). The Col/Tra/Gel showed continuous and biphasic release of protein drugs for up to 9 days. On SC administration, the mice demonstrated the highest tumor growth inhibition in comparison to other groups. The histological and immunohistochemical analysis validates the gel-induced digestion of collagen and apoptotic cell death. The experimental results prove that the prepared hydrogel system for breast cancer triggers the degradation of intra-tumoral collagen, promoting the penetration and retention and finally improves the antitumor efficacy of trastuzumab [84].

Hydrogel systems can also be utilized for ocular drug delivery due to their ease of application low irritation and biocompatibility. Cocarta et al., developed a hydrogel implant for chemotherapeutic drugs for localized sustain drug delivery for avoiding retinal and systemic toxicity with effective control of tumour growth while preventing relapse. The developed hydrogel implant consists of two layers firstly inner hydrophilic layer consisting of 2-hydroxyethyl methacrylate (HEMA) and an outer hydrophobic layer consisting of 2-ethoxyethyl methacrylate (EOEMA). Hydrogels were prepared from polymer which was synthesized by crosslinking free radical polymerization by photoinitiation. Topotecan and vincristine sulfate were loaded in the hydrophilic layer by immersing swollen hydrogel into the drug solution at 4 °C. In-vitro release study exhibited a biphasic release profile with 60% drug release in the first 5 h for topotecan followed by sustained release up to 20 days, whereas in the case of vincristine 80–90% burst release was observed in the first 5 h consequently 100% release was observed after 24 h. In-vitro cytotoxicity studies of hydrogel demonstrated the cytotoxic effect of topotecan for up to 2 days and vincristine sulfate for up to 6 days in retinoblastoma cell line [85].

Hydrogel systems can be an excellent choice for drug delivery to post-surgical cavities as the cavity is irregular in-situ forming hydrogel possesses excellent malleability, minimum invasive process along shape adaption function [86]. Zhuang et al. designed an injectable hydrogel of gemcitabine (GEM) and doxorubicin (DOX) to prevent recurrence of local cancer. The rapid hydrogel formation at the post-surgical cavity was observed by injecting the mixture of aldehyde hyaluronic acid and carboxymethyl chitosan. DOX was conjugated with aldehyde hyaluronic acid gel via Schiff-base reaction (8% w/v in PBS) and hydrophilic GEM was dissolved in carboxymethyl chitosan, dual drug-loaded gel was obtained by mixing these drugs-loaded gels at room temperature. Complete release of GEM was observed in 3 days whereas only 3.85% DOX was released in 14 days in in-vitro drug release studies. A synergistic anti-cancer effect was observed on 4T1 cells. Dual drug-loaded hydrogel was implanted into the post-surgical cavity, and prohibition of cancer recurrence and distant lung metastasis was observed in comparison to single drug-loaded gel [87].

Peptides possess good bioactivity and specificity for clinical application, but due to rapid degradation and shorter half-life hinder the peptide activity in-vivo. Li et al. developed a novel peptide hydrogel of calcitonin gene-related peptide (CGRP) by photocrosslinking of hyaluronic acid (HA). The novel peptide hydrogel involved the formation of covalent bonds between hyaluronic acid and CGRP for achieving sustain release of the peptide to promote sustained bone regeneration. The prepared hydrogel promoted cell proliferation and good biocompatibility with bone marrow stem cells (BMSCs). The increase in the level of expression of osteogenic genes and proteins was observed to up-regulate osteogenic differentiation capabilities in in-vitro osteoblast differentiation assay. In-vivo study demonstrated an acceleration in new bone formation by the protein-loaded hydrogel [88].

### Oleogel formulation

**Fig. 3:**
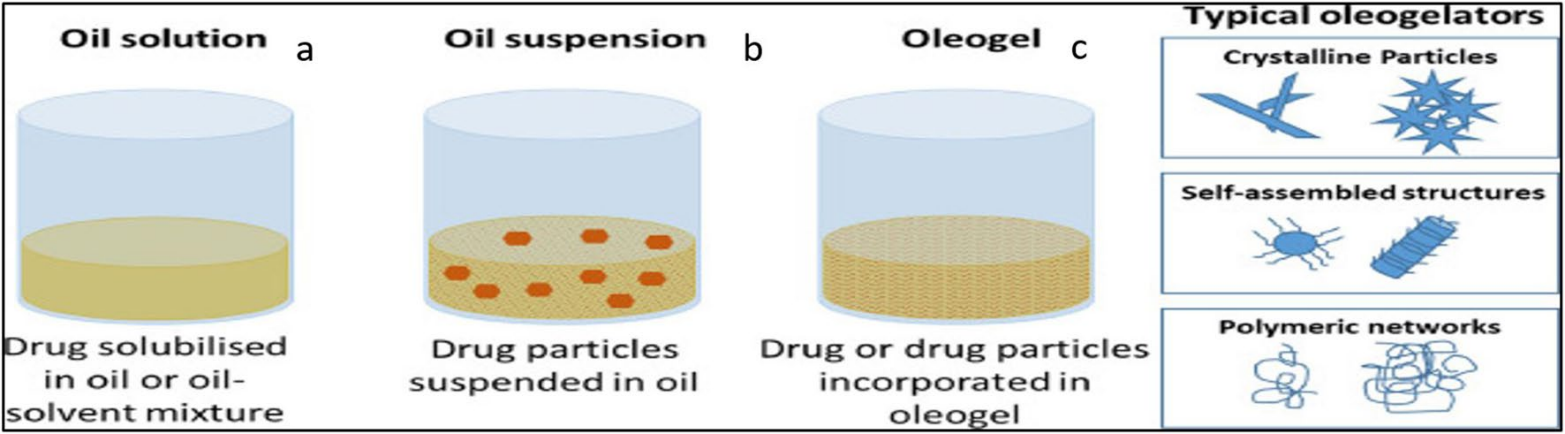
Visual representation of (**a**) Oily solution, (**b**) Oil suspension, and (**c**) Oleogel figure reproduced, with permission, from [5]

Oleogels are semi-solid systems consisting of oil as a liquid constituent, immobilized by the gelator into a continuous phase [23]. Oleogel formulations have been explored for drug delivery via various routes such as injectable, topical, and oral [89–91]. Oleogels are structurally different from oily solutions or suspensions (Fig. 3). Usually, oleogels are delivered by SC or IM route. Formation of oleogels involves the immobilization of oil within spaces of a 3-dimensional network created by gelator molecules due to physical interaction or chemical cross-linking [62]. Oleogel prepared by chemical cross is not often used in drug delivery. The formation of oleogels via physical interaction is solely dependent on supramolecular self-assembling of oleogelators stabilized by non-covalent interactions namely H-bonding, π-π stacking, and van der Waals interactions [62, 92].

The formulation components of oleogel formulations include oleogelator, oil carrier, and API. The concentration of the gelator should be less than 15% to maintain the injectability. The solubility of the gelator in oil is the most important as it has a direct impact on the stability of oleogel. Based on the molecular weight oleogelators have been classified into low molecular weight oleogelators (LMOGs) and high molecular oleogelators (HMOGs). LMOGs include waxes, alcohol, fatty acids, and monoacylglycerols [93], whereas HMOGs examples include polymers, proteins, and gums that form gel in oil-based systems. Based on the required properties of the final formulation, the selection of components and formulation technique is made. The Oleogel system offers several advantages over emulsion-based systems such as

- Stabilization of emulsion in gel
- Undergoing shear thinning behaviour
- Thermo-reversible nature
- Allows manipulation of drug release by changing the concentration of gelator or ratio of oil to gelator
- Longer shelf life due to absence of aqueous components
- Biocompatible

Vegetable oil-based oleogel systems have been reported to incorporate both hydrophilic and lipophilic drugs. The study reported by Macoon et al. involved the gelling of emulsion containing two hydrophilic drugs (timolol maleate and metformin hydrochloride) for ophthalmic delivery. Ethyl cellulose was used as a gelator and soybean oil as the oil phase with tween 80 as surfactant. Two systems where prepared firstly particulate system in which the drug was added in the oil phase above its solubility to form a dispersion, whereas in the other system both the drugs were dissolved in an aqueous system and W/O emulsion was formed which was further gelled with ethyl cellulose. In-vitro drug release was performed for both the systems, in the particulate system metformin hydrochloride demonstrated slow release for up to 120 h whereas gelled formulation showed sustained release for up to 1400 h. For timolol maleate up to 400 h, the drug release from the particulate system was observed which was increased up to 1000 h by increasing the drug concentration in the system, however gelled system showed drug release up to 2200 h. The semisolid nature of prepared oleogel allowed the injection of formulation using a 20 G needle forming a rod-shaped implant post-injection [94].

To overcome the syringeability issue of oleogel systems, the addition of an organic solvent for example NMP (n-methyl pyrrolidone) and DMSO (dimethyl sulfoxide) for inhibiting gelation and facilitating injection has been investigated [95]. Due to the biocompatibility and hydrophilic nature of NMP, it is commonly used in oleogel systems. Owing to the hydrophilic nature of NMP diffusion into surrounding tissues on administration occurs leading to the conversion to gel form. The addition of these cosolvents inhibits the gelation process partially or completely maintaining the liquid state of formulation for injection. NMP was shown to successfully reduce the gelation of N-stearoyl-L-alanine methyl ester (SAM) containing gel allowing subcutaneous injection in rats [89]. For example, Vintiloiu et al. prepared rivastigmine oleogel using N-stearoyl-L-alanine methyl ester (SAM) gelator in safflower oil for subcutaneous injection. Oleogel exhibited lower initial burst release (< 15%) in 24 h. The in-vitro release and in-vivo pharmacokinetic study confirmed the application of SAM oleogel for obtaining sustained drug release, therapeutic concentration was maintained for up to 11 days after a single injection at 10% concentration of gelator (SAM) [89].

The application of oleogels for delivering local anaesthetic drugs has been explored by Wojtalewicz et al., where bupivacaine oleogel was formulated using three different glycerides i.e., tripalmitin, glyceryl monostearate (GMS) and geleol and medium chain triglyceride oil was used as oily vehicle. In the first 30 h, geleol containing oleogel showed 25.28% ± 0.93% whereas GMS and tripalmitin showed 21.27% ± 0.97% and 19.76% ± 0.39%. At the last timepoint 582 h 90% drug release was expected, for geleol, 97.30% ± 0.29% drug release was observed whereas for GMS and tripalmitin, it was 90.02% ± 0.20% and 91.23% ± 0.24%. The % bupivacaine released from GMS and tripalmitin was lower compared to geleol oleogel. GMS and geleol formulation required higher injection forces than the tripalmitin (82.5 ± 4.6 N and 112.9 ± 12.3 N). Overall GMS containing oleogels showed superior oil retention, injection forces, and thermal stability, in addition to this in-vivo studies demonstrated GMS oleogel provided an anaesthetic effect for 4 times and 2 times longer duration than the bupivacaine HCl and bupivacaine-loaded plain MCT oil. Another observation from the study was the prolongation of drug release can be achieved by increasing the viscosity of vegetable oils [96]. The nanoparticle-loaded oleogel was developed by Narayanan and the team to prolong the release of tenofovir alafenamide (TAF) a hydrophilic drug. Firstly, tenofovir-loaded chitosan nanoparticles were prepared by spray drying. Oleogels were prepared using ethyl cellulose as a gelator and sesame oil. TAF nanoparticles showed initial burst release (< 20%) in 2 h with 100% release observed in 26 h Whereas, extended release of tenofovir alafenamide (56%) was observed from oleogel for 16 days [97].

Lampp and team explored the application of radical oleogel for the diagnosis, as the quantification of molecular oxygen in tissues has a probability of understanding and diagnosing diseases and developing a therapy. Lampp et al. developed a radical containing in-situ oleogel for oxygen measurements for prolonged time with electron paramagnetic resonance. A radical containing oleogel was prepared using 12% of 12-hydroxy stearic acid (12-HSA), N-methyl pyrrolidone (NMP), and Isopropyl myristate (IPM). Due high solubility of oxygen in the oil phase increased the oxygen sensitivity. The lifetime of radicals in the tissues was extended from 10 min to 21 days simplifying the study with the increase in the series of oxygen measurements [98].

### Aqueous suspensions

Aqueous suspensions are another interesting approach in long-acting injectables for prolonged therapeutic delivery in neurodegenerative diseases. In this, the crystalline drug or drug complexes of size between 100 to 1000 nm are suspended in aqueous media and are generally administered through an intramuscular route [99]. Invega Sustenna^®^, Xeplion^®^, an intramuscular extended-release injection of paliperidone palmitate was approved to manage schizophrenia in 2009 [100]. The clinical intervention of the drug formulation (50 mg or 100 mg eq) vs placebo was given to *n* = 197 patients. The results showed a notable improvement in the mean (SD) total scores of the Positive and Negative Syndrome Scale (PANSS). In conclusion, the authors emphasized that the Paliperidone long-acting injection (PLAI) was efficacious in both short-term and maintenance treatments [101]. similarly, Eli Lilly marketed the antipsychotic long-acting injection Zyprexa^®^ Relprevv™ containing olanzapine pamoate salt in the year 2010. Lindenamyer et al. summarized the data from the 8-week clinical trial study performed on 404 inpatients in a randomized design. The long-acting injectable formulation was effective against oral formulation with an improved side effect profile. However, Zyprexa^®^ Relprevv™ administration can be challenging due to the presence of salt form for example if given intravascularly large amount of the drug may get released, causing an overdose [102].

Mandal and co-workers explored the long-acting potential of tenofovir alafenamide (TAF) and emtricitabine (FTC) by using PLGA _75:25_ polymer [103]. Both the hydrophilic drugs were nanoencapsulated by using water-in-oil-in-water (W-O-W) emulsion method. The prepared nanoparticles were evaluated for its long-acting potential by injecting subcutaneously in humanised mice (Hu). The prepared nanoparticles were compared against the TAF + FTC solution at dose of 200 mg/kg each. The prepared nanoparticles showed enhanced tissue antiretroviral assimilation compared to plasma in Hu-CD34-NSG mice. The same dose was given to the Hu-BLT mice group. On the 21st day post treatments 100% infection was observed in mice treated with TAF + FTC solution, whereas, in TAF + FTC nanoparticle administered group, significant (*p* = 0.0002) protection from HIV-1 with 80% protection on day 4, 60% on day 7 and day 14 was observed. This result demonstrates the long-acting potential of the prepared nanoparticles for hydrophilic drugs [103].

Milling is the most common technique in the development of aqueous suspensions however, it presents some challenges like scaling up and maintaining aseptic conditions during manufacturing, and initial burst release [104]. These drug products can be useful in some disease conditions, but more extensive screening in validating in-vitro release profile and lag period can be beneficial in quality control.

## Factors acting designing of long-acting injectables

To determine the suitability of formulating into a long-acting injectable, the drug molecules must meet specific criteria, which have been discussed thoroughly in the subsequent section.

### Physicochemical properties of drug molecules

The physicochemical properties of the drug molecule such as particle size, water solubility, partition coefficient, and half-life are the vital parameters in the formulation of LAIs, as it has a direct impact on the release of drugs from the systems as well as the selection of the type of system.

#### Particle size

The particle size is a critical factor as it affects the injectability and dissolution of drug molecules [105]. Smaller particles ease the administration through larger gauges ideally preferred for SC injection self-administration, nevertheless, particles of size less than 10 µm are susceptible to rapid phagocytosis and lead to quick clearance from the system [105]. Dissolution of smaller particles will be faster due to the large surface area leading to a reduction duration of therapeutic concentration. Conversely, larger particles will dissolve slowly providing sustained release. Particle size has an impact on the pharmacokinetic properties of drugs as well. The study conducted by Ho et al. investigated the effect of the lipidic pro-drug of entecavir (entecavir-3-palmitate) nanosuspension particle size on its pharmacokinetics entecavir-3-palmitate particles of various diameters (0.8 – 22.6 µm) exhibited different dissolution profiles as well as local inflammatory responses at the site of injection. The particles of size 0.8 µm showed greater systemic exposure compared to 6.3, 15.3, and 22.6 µm particles, in addition, the particles of size 0.8 µm showed higher inflammatory lesions after 3 days, fewer infiltrations of fibroblasts near the depot after 4 weeks. On the contrary for higher particle size increase in drug deposition at the depot site was observed over 4 weeks [106].

#### Partition coefficient

Partitioning of drugs from oily vehicles to tissue fluids is mainly dependent on the lipophilicity of drugs. This partitioning of the drug is the rate-determining step controlling the percentage of drug absorption in the systemic circulation after intramuscular injection [107, 108]. In favor of this, a linear correlation has been reported between the log absorption rate and the log oil-water distribution coefficient [109, 110]. Hence the rate of drug release from vehicles can be tailored by changing the lipophilic chain in prodrug or vehicle choice [111].

#### Water solubility

Each formulation design requires adequate solubility in water or organic solvents to aid in formulation development, where aqueous soluble drugs are preferred for depo foam technology whereas lipophilicity is desired for oily solutions or aqueous suspensions [112]. Drug candidates with poor water solubility are difficult to deliver via oral or intravenous route but in the case of long-acting nanosuspensions, it is a desirable characteristic [113]. Many hydrophilic drugs were modified into lipophilic/hydrophobic pro-drugs to develop long-acting injectable suspension examples are listed in the oily suspension section.

#### Half-life of drug molecule

Drugs with short half-life require repeated administration to maintain the plasma concentration even after dosing with conventional parenteral forms i.e., IV route. Drug with longer half-life is preferred for developing long-acting injections which will maintain therapeutic concentration for a longer time, drugs with ultra-short half-life cannot be good candidates for LAIs. E.g., The half-life of native glucagon-like peptide 1 (GLP 1) is 1 to 2 min after IV administration, whereas its analogue exenatide has a half-life of 2.5 h after subcutaneous administration [114]. It is not possible to design LAI for GLP-1 but an exenatide-loaded microsphere formulation (BYDUREON) is designed and marketed for once-a-week administration [115].

#### Dose

Potent drug molecules are ideal candidates for LAIs, whereas drugs that require large doses are difficult to formulate into LAIs. Peptide molecules have low oral bioavailability, but they are very potent molecules with low doses which makes them good candidates for LAIs.

### Route of administration

Most LAIs are delivered by IM or SC route, the selection of route depends on the volume of formulation that needs to be administered. For the intramuscular route volume of up to 5 ml can be delivered, whereas for the subcutaneous route volume is limited to 1 to 2 ml [116]. Injection volume is an important parameter to consider in the case of high-dose drugs. The site of injection can also influence the drug release, injection into highly vascular tissue can lead to faster drug release compared to that to less vascularised tissues where drug release will be slower.

### Patient variability

Various patient-related factors such as age, sex, disease condition stage, and metabolism state can also impact the drug release from LAIs.

## Mechanism of drug release

Based on the type of the long-acting system each follows a different mechanism for the release of drug from the formulation. The mechanism followed by each system is discussed in detail in the following section.

### Multivesicular liposomes

**Fig. 4:**
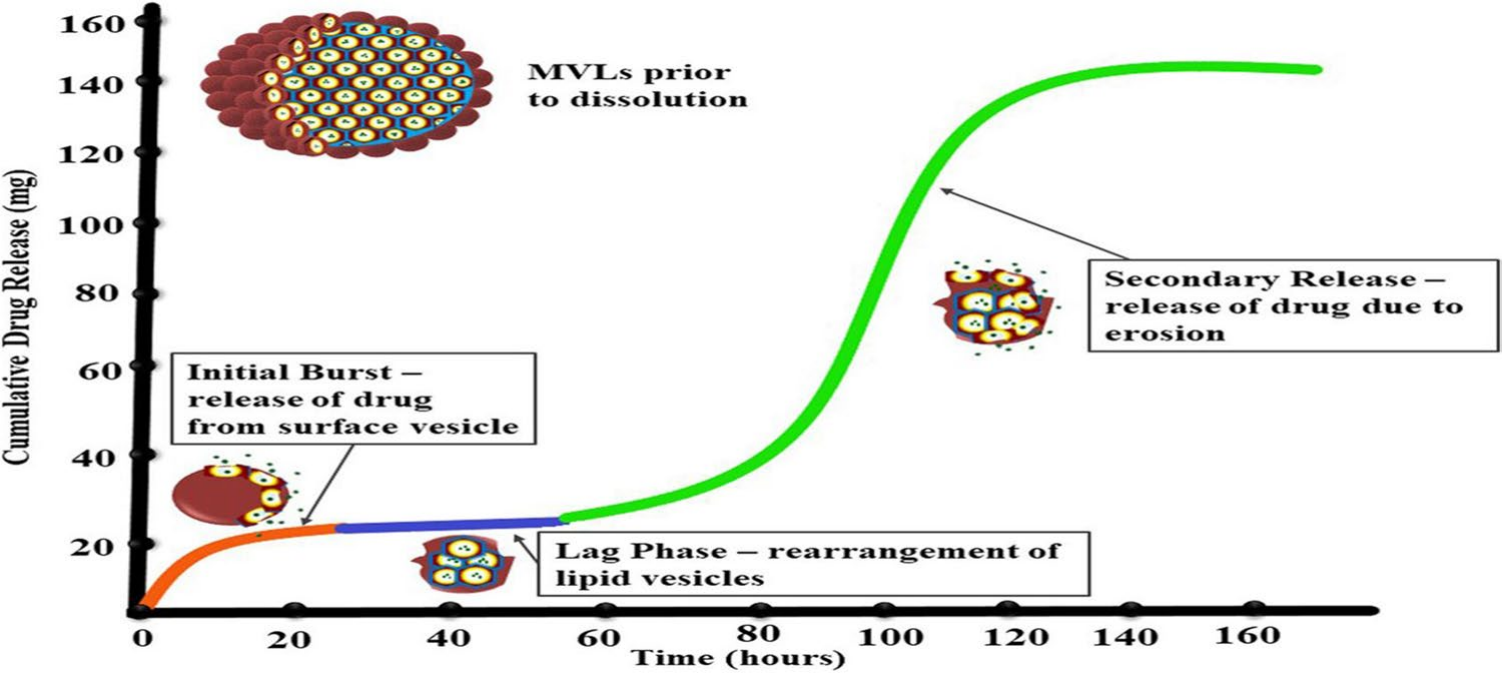
The illustration of the different release patterns of drug release for MVLs figure reproduced, with permission,from [117]

Various factors affect the release of drugs from the multivesicular liposomes such as physicochemical properties of the lipids used, particle size, pH, osmolarity, and temperature [27]. The drug release from MVLs occurs due to a combination of diffusion of the drug from the liposomal membrane and erosion of MLV [33]. The experiment conducted by Manna et al., where drug release from bupivacaine-loaded MVL particles was studied using a modified USP type II apparatus where a triphasic drug release pattern was observed. Firstly, initial burst release was observed from the drug present on the surface as well as the free drug present. Secondly, the lag phase with a slow release of the drug due to the drug of the lipidic membranes. Lastly, the secondary release results from the erosion of MVLs from lipid hydrolysis [117] (Fig. 4 illustrates the different release patterns followed by MVLs).

### Microspheres

The polymer used for preparing microspheres affects the release kinetics of the drug [118]. Depending upon whether the drug is encapsulated or embedded within the matrix the drug release mechanism follows diffusion/dissolution or erosion kinetics. When the drug is dispersed in the matrix system, in addition to the properties of the polymer other factors such as drug properties, and release media also affect the dissolution and release behaviour of the drug. The diffusion of the drug occurs via pores formed by the water molecules or via intact polymer. The presence of water causes the swelling of the polymer which increases the diffusion coefficient of the drug followed by erosion of the polymer [119]. Whereas in the case of encapsulated drug molecules, the coating of the microspheres will dissolve itself or swell in the release media which acts as the permeable membrane for drug molecules [120].

### Hydrogel

Hydrogel systems have been used for various applications such as controlling drug release, tissue regeneration, and localized drug delivery. The ratio of formulation component of the hydrogel can be adjusted for optimizing the drug release and the porous nature of hydrogel allows the loading of drug molecules of various molecular sizes [78]. The release of encapsulated from hydrogel occurs due to passive diffusion or degradation of hydrogel [121]. The degradation of hydrogel occurs due to water or enzyme hydrolysis. The release rate can be determined by studying the gel degradation rate, drug-polymer interaction, and the hydrophobicity and molecular weight of the drug. Consecutive degradation of hydrogel occurs with the slow release of the drug molecule [13]. In the case of diffusion-controlled release mechanism extremely high burst release is observed up to 50% with short release duration which can be extended by inducing electrostatic interaction or hydrophobic interaction with drug and polymer which will extend the release profile up to 2–3 days [122]. The release profile of up to 1 week can be obtained by forming a cleavable covalent linkage or degradation-controlled mechanism [122]. The combination of hydrophobic interaction and degradation-controlled mechanism can be used for extending drug release for a longer duration e.g., Pluronic F127 hydrogel was prepared by embedding PLGA encapsulated VEGF which showed sustained release up to 60 days from the gel [123].

**Figure:**
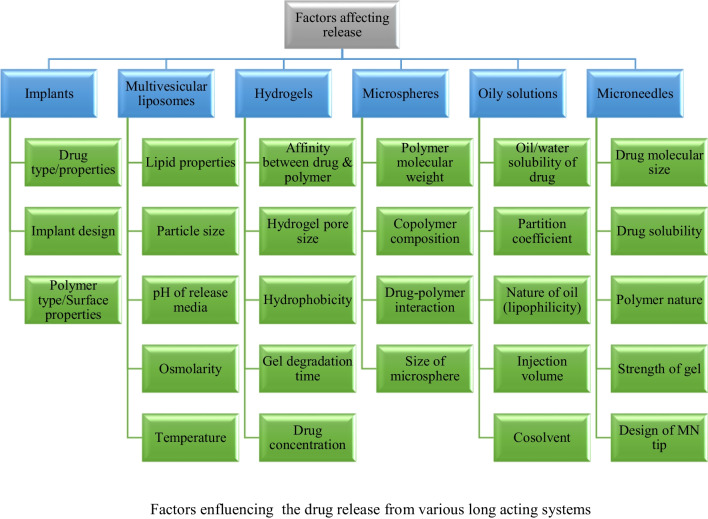
Factors enfluencing the drug release from various long acting systems

## Challenges with the development and use of long-acting injectables

### Issues related to development and manufacturing

The development of long-acting injectables for chronic diseases that require lifelong treatment is becoming popular to increase patient compliance and treatment adherence. The potential of LAIs to deliver high and low molecular weight compounds for prolonged periods makes them interesting candidates in drug delivery meanwhile it also invites some major challenges around it. For example, delivering large molecules such as peptides, phytoconstituents, synthetic large molecules, and complex mixtures of drugs can undergo physical and chemical changes after loading into long-acting formulations. Moreover, these high molecular weight compounds show high-order structural configurations, which makes the formulation synthesis more complicated. Opting for different formulation steps like using the right solvent or buffer, purity of excipients, and need for concentration adjustments to avoid inhomogeneity [124]. Also, to increase drug loading into these long-acting formulations strategies like the usage of ester-capped PLGA to prepare NPs [125], incorporating more compatible excipients (solvent or cosolvents) [126], changing drug physicochemical properties (converting salt form to free base/acid form) [127], adjusting the particle size of the carrier, and opting for the best-suited preparation method (Microfluidics, single or double emulsion, spray drying, or complexion technique) [128]. However, storage methods like freeze-drying of the proteins and other high molecular compounds can cause ice-crystal transformation and sudden pH change leading to reduced stability and efficacy [129]. Light exposure, mechanical stress, aggregation, fragmentation, and unfolding also contribute to the difficulty in formulating these drug products [130]. Similarly, LAIs containing low molecular weight compounds also exhibit several challenges including safety and efficacy.

The manufacturing process and different components used in LAI development can play a crucial role in modifying the pharmacokinetic (PK) profile of these drug products, for instance using different polymers and solvents in the synthesis process can alter the surface morphology and pore size of drug-loaded microspheres, and finally changing release profile from these nanoparticles [131]. Various concepts have been proposed to understand the burst release from these pharmaceutical products. One such study by Kim et al. demonstrated the effect of different process conditions on the initial burst release. the findings illustrated less initial burst release from the particles produced by vacuum drying at room temperature possibly due to the drying temperature within the glass transition temperature [132]. Similarly, some other concepts to understand the effect of different polymer-to-drug ratios, the physiochemical nature of drug molecules, and process parameters on the final products have been explored. The syringe ability and injectability are other critical parameters that need to be considered while manufacturing LAIs, which are dependent on particle size, shape, viscosity, and final formulation concentration [133, 134].

### Patient acceptability and injection-related issues

Patient acceptability and compliance with long-acting injectables are very important and should be considered in the design and development of these LAIs. The overall concept of acceptability is not only an attribute of intervention but also a subjective evaluation by the recipient of therapy and the healthcare professional delivering it. However, evaluating the acceptability can be performed at different time points such as pre-, throughout, and post-intervention [135, 136]. Similarly, patient acceptability in injectable drug products can be defined as “the overall ability and willingness of the patient to use a medicinal product as intended and its caregiver to administer the medicine as intended” [137]. LAI delivery in elderly patients could induce anxiety and can show immediate or delayed hypersensitivity to the needles. Moreover, patient gender, low body mass, and multiple disease conditions can also reduce the acceptability of LAIs as a therapeutic intervention [138].

The ability of LAIs to deliver drugs for prolonged periods can be influenced by physicochemical properties like distribution, metabolism, and clearance after administration of these molecules, finally increasing therapeutic outcomes. However, sometimes these drug products can show deviation from the predicted PK profile and hence reduce clinical acceptability. For instance, highly potent molecules with low molecular weight can diffuse rapidly from the site of the administration to surrounding tissues and can cause local or systemic toxicity. In contrast, higher molecular weight drugs like biologics have relatively low clearance and distribution, and their ability to form inter and intramolecular bonds may significantly influence the therapeutic plasma concentration and require dose titration [139–141]. Also, the post-administration monitoring of LAIs is required to avoid toxicity due to a large amount of API present in the drug delivery system which may increase the healthcare burden [142].

The formulation components used in injections can exhibit immunogenicity for example PEG used in nanoparticulate formulations, is associated with an antibody-specific immune response. Li et al. studied the immune-responsive activity of protein-grafted poly(ethylene glycol) (PEG) in the C57BL/6 mice model. The results from the study illustrated a hapten-like PEG-specific antibody immune response confirmed via enzyme-linked immunosorbent assay (ELISA) and surface plasmon response (SPR) tests, which can be lethal to humans (Li 2018). The differences in the physiology of animals and humans may trigger different immune responses in testing. However, various natural and synthetic polymers are generally regarded as safe (GRAS) to use in humans for prolonged periods. Apart from this, the IVIVC relationships are hard to establish in LAIs due to their complexity in depicting in-vivo behavior from the in-vitro release profile [143]. The lack of an ideal in-vitro release method and product composition variability also contribute to the deflection from the in-vitro-in-vivo correlation [144].

### Regulatory and cost-related challenges

Currently, in the long-acting medication realm, antipsychotics have gained popularity in recent years and are majorly prescribed neurotherapeutics with a worldwide market of USD 16.14 billion in the year 2023. Following this trend, the market is projected to increase to USD 26.48 billion by 2035 going with a CAGR of around 5.4. Long-acting injectables provide various clinical advantages but the development of their generic equivalent products may present challenges due to their complexity, the presence of intellectual property, and the degree of ease of use of the manufacturing process [145]. The debate over the cost of LAIs in short-term treatment can increase the economic burden to some extent in comparison to the standard of care, whereas their long-term treatment in various disease conditions can be cost-effective, especially in neurological conditions like Alzheimer’s, schizophrenia, and Parkinson’s [146].

The approval process for LAIs requires some degree of correlation using bioequivalence and bioavailability (BABE) to establish their therapeutic effectiveness. The guidelines for Oral Generic Products are well-defined in comparison to polymeric long-acting drug products [145, 147]. This may be due to the lack of a standard In-vitro release model defining the pharmacokinetic profile of the API from the polymer matrix [148]. However, the physiological based pharmacokinetic (PBPK) modeling is very important for regulatory approval like the Food and Drug Administration Office of Generic Drugs (OGD) in assessing the bioequivalence of generic drug products. Also, its use can decrease the number of clinical trials required in the approval process. Furthermore, studies have illustrated that using USP type 4 apparatus for predicting in-vitro release gives a level A IVIVCs, defining its promising potential in long-acting drug product testing [149].

Despite the challenges, these long-acting medications have gained popularity due to their advantages like less frequent delivery, low relapse rate, patient compliance, improved PK profile, and less drug abuse, especially in neurological disorders. More pharma companies (for example Eli Lilly [150], Johnson & Johnson [151], Zogenix and Durect [152], Pfizer [153] and GSK [154]) are investing in the research and development of these LAIs, and this will show significant growth in the near future [155].

## Preclinical application of long-acting injectables in neurodegenerative disease

### Lipid-based LAIs

Dudhipala et al. fabricated two hydrogels containing nanostructured lipid carriers (NLC) and solid lipid nanoparticles (SLN) nanoparticles (NP) loaded with ropinirole for treating Parkinson’s disease (PD). Both the optimized formulation of NLC and SLN NPs were characterized using particle size, zeta potential, PDI, drug loading and encapsulation efficiency, in-vitro release, ex-vivo permeation, and in-vivo studies. The results showed a size lower than 250 nm, a PDI lower than 0.25, and a zeta potential higher than -25 nm ideal for topical delivery. Drug loading and encapsulation efficiency for both formulations were found to be more than 7% and 97% respectively. Both NPs were then loaded into a gel system of topical delivery, and the cumulative release from SLN was 84.3%, and from NLC was 92.3%, slightly higher in the case of NLC NPs over 24 h. The ex-vivo permeation studies using rat skin displayed a flux value of more than 2 folds in both formulations. Also, the in-vivo studies in Wistar rats showed a significantly increased Cmax value, with higher bioavailability almost 2-fold compared to the control formulation. Moreover, the pharmacodynamic evaluation revealed the restoration of different biochemicals in rat models with induced PD. However, further exploration in clinical studies will unravel the full potential of the formulation [156]. Another microemulsion gel formulation containing rasagiline mesylate, an irreversible MAO-B inhibitor for PD, was generated by the ternary phase method. The mean globule size of the emulsion was 171.8 nm with a PDI value of 0.2 and -2.13 mV zeta potential. The pH of the gel formulation was well within the human tolerance range (between 5.5 and 6.5) and a high encapsulation efficiency of around 100% was observed. The data from the rat skin permeation studies showed a high flux value of 83.47 μg/cm2/h in 24 h. In-vivo, efficacy data also illustrated around a 2.5-fold increment in motor function tests in comparison to oral solution [157].

Topal et al., apolipoprotein-labeled SLNs were synthesized to effectively deliver donepezil and rhodamine B for the treatment of Alzheimer's disease (AD). The nanoparticulate SLNs were developed from homogenization followed by the sonication technique and surface modified with ApoE. In characterization studies, the size of NPs was around 147.5 nm with a PDI of 0.22, and a zeta potential of -9.62 mV. In addition to this, the entrapment efficiency for donepezil and rhodamine B was 86.44% and 94.90% respectively. In-vitro, cumulative release data suggested that 50% drug was released from surface-modified SLNs after 72 h. Similarly, the in-vitro permeation studies using fluorescein dye illustrated a 3.2-fold higher permeation with surface-modified NPs. The prepared particles were stable and increased the uptake behavior of SLNs, which can be used in the effective management of AD [158]. In another work by Bhandari et al., a lipid-based nanocarrier system using chitosan and soy lecithin, where homogenized probe sonicator and desolvation processes were utilized. Donepezil-loaded NP particle size, PDI, encapsulation efficiency, and zeta potential were measured as 278.86 nm, 0.09, -5.53 mV, and 8.77% respectively. The percentage cumulative release from the gel was observed as 36.33% after 45 days and Higuchi was the best-fitted release model. Moreover, the cell viability studies in the L929 cell lines revealed more than 80% viable cells in all samples showing safety and nontoxicity. The mucoadhesion studies in the porcine stomach by Bradford assay suggested no change in mucoadhesiveness after drug loading [159].

Interestingly a combination of memantine and tramiprosate was loaded into solid lipid nanoparticles using the homogenization-ultrasonication method for better brain delivery [160]. There was high cell viability in drug-loaded SLNs when compared with pure drug and amyloid aggregates in the MTT assay. This was probably due to the toxicity of pure drug and Aβ aggregates in cell lines which was resolved with loading into nanoparticles. The optimized particles revealed a size of 157 nm, and a PDI of 0.149 respectively. The drugs were released slowly following the Higuchi model from the nanoparticles for up to 48 h. Similarly, in-vivo studies after intraperitoneal injection showed sustained release with an improved PK profile. Pharmacodynamic studies like Morris water maze (MWM) in AlCl3-treated rats showed a significant reduction in Aβ protein in the brain. The sustained release of drugs for longer periods benefited in treating diseased rats and can be translated into clinical trials. In another study, NLCs were synthesized from a solid-phase peptide synthesis technique [161]. Tacrine, a drug previously approved by the FDA for AD, was loaded into NPs. The study aimed to improve the pharmacokinetic profile along with safety and prolonged release. The results reflected sustained release with 60% drug diffused after 72 h. Also, the safety and low toxicity were confirmed by MTT and sulforhodamine B (SRB) assay.

### Polymer-based LAIs

Recently, the prolonged delivery of therapeutics has resulted in the development of polymeric long-acting injectables as an alternative to conventional oral delivery systems. These advanced drug delivery systems have shown promising results in improving pharmacokinetic profiles and different physiochemical characteristics such as solubility and stability. We have compiled preclinical data as discussed below:

Salles et al. [162] developed a polylactic acid (PLA) based polymeric delivery system loaded with liraglutide, a glucagon-like peptide for neuroprotective action in AD. The gelatin-coated fibers were prepared by electrospinning technique and were given subcutaneously for controlled release. This fiber biodevice was subjected to in-vitro oxidative (H2O2) DNA damage studies in human neuroblastoma cells (SH-SY5Y) and results revealed no neurotoxic effects on cells after biodevice application. Furthermore, biodevice activated the antiapoptotic Akt pathway leading to cell survival. The in-vivo release studies from the implant showed an initial burst release followed by a controlled release for up to 14 days. Similarly, these fibers significantly reduced the plaque load in APP/PS1-treated animals, increased neuroblast activity, and reduced the astrocytic activity due to prolonged drug release. These findings concluded that reducing the dosing frequency can enhance the efficacy of the treatment regimen significantly [162].

Microspheres nanoparticle is another approach to deliver therapeutic for prolonged periods ranging from a few days to several months. Kim and subordinates prepared porous PLGA microspheres coated with alginate utilizing the spray ionotropic gelation method. The microspheric formulation was subjected to characterization studies and results suggested that with an increase of sodium alginate to PLGA ratio, the increase in particle size was observed possibly due to the thicker coating. Also, the span values were between 0.67 to 1.23 showing a narrow distribution. However, the effect of pore size on encapsulation efficiency showed a positive relationship, where with bigger pore sizes the % EE was 83.2% and loading capacity was 5.25%. the in-vitro release from the microspheres with higher coating revealed slower drug release in the first 8 h with a value of 47.3%. Moreover, the pore-closed formulation depicted an extended-release with almost 2.7 times longer release in-vivo pharmacokinetic studies. The longer release and less frequency make these microspheres a better choice and can show improved clinical attributes [163]. Subsequently, to reduce the burden of AD, anti-amyloid nanoparticles were prepared by Carradori et al. and tested in a transgenic mice model for their efficacy. The nanoparticles were synthesized by Bio-Sav ligation technique using pegylated (P(HDCA-co-MePEGCA)) and exhibited a particle size of 182 nm and PDI of 0.22. these NPs had a negative zeta potential between -20 to -30 mV. The PK and bio-distributional studies revealed high blood circulation time due to conjugation with PEG, however, a smaller number of NPs were diffused into the brain with approx 0.5%. The NOR behavioral study in the treated transgenic mice model showed a restored memory function in comparison to mice receiving PBS. Furthermore, the ELISA assay results on brain and plasma homogenates for amyloid protein detection showed a 20% reduction of these biomarkers in the treatment group, the findings were further confirmed using western blotting. In conclusion, this study supported the “sink effect” hypothesis and can be useful in reducing beta aggregates [164].

Interestingly, a subcutaneous depot system of PLGA/PLA containing rivastigmine was formulated for the safe and effective treatment of dementia related to AD. The authors prepared a phase-sensitive formulation using homogenization and sonication and optimized it for improved in-vitro/in-vivo activity. Optimized PLGA in benzyl alcohol/benzyl benzoate system showed low initial burst and controlled release up to 14 days. The AChE studies depicted an enzymatic inhibition even after complete drug release from the formulation. Additionally, the histopathological biocompatibility evaluation suggested exceptional safety with resolved signs of inflammation and vasodilation of the formulation after application. Altogether, it can be concluded that the polymeric depot was effective as well as safe to use and can be promising in developing treatments for AD [165].

In another study lopez-cano and co-workers synthesized a triblock copolymer thermo-responsive polymeric hydrogel system containing dexamethasone and ketorolac. Ring-opening polymerization technique was used to formulate the intravitreal injection for the management of glaucoma. The micellar size range of both drug formulations was between 23.02- 34.8 nm. In-vitro, release studies revealed the sustained release of drugs for more than 50 days following the Korsmeyer-Peppas model. Moreover, cell in-vitro toxicity studies predicted a high tolerance between toxic effects and concentration. The oxidative stress (H2O2) MTT assay showed high viability values of more than 80% with four-fold higher values in the treatment group. Also, the anti-inflammatory activity of the hydrogel was observed in TNFα values with dexamethasone and ketorolac [166]. In a similar study, Adent et al. developed a thermosensitive intranasal polymeric gel and liposomal formulations for better brain bioavailability in AD. The thin lipid film hydration method was utilized for liposomes, whereas the cold method was used for gel synthesis. The results from characterization studies including particle size, PDI, zeta potential, and encapsulation efficiency were 114.9 nm, 0.048, -11.2 mV, and 11.1% respectively. Subsequently, the sol-to-gel temperature was 34.5 °C and pH of 6.5. The in-vitro release studies in nasal simulated fluid revealed sustained release kinetics with T50% of 60 min. In conclusion, the developed liposomal gel has high efficacy and can be optimized for better delivery [167].

Jiang and colleagues prepared an in-situ gel loaded with rasagiline mesylate microspheres for PD treatment. The highlight of the study was to develop an optimized formulation using an emulsion-solvent evaporation technique. Optimized microspheric formulation showed a size of 63.68 mm, drug loading of 30.12%, and encapsulation efficiency of 89.88%. The release profile of the NP-loaded gel depicted an 85% cumulative release for 60 days with a controlled initial burst following the Korsmeyer Peppas model. Additionally pharmacokinetic evaluation sprague dawley (SD) mice model suggested that the Cmax/Css value of 7.06 showed lower initial burst release, also the half-life time from the gel was 25.6 days. High treatment efficacy was observed with high-dose gel implant in the pharmacodynamic studies and restored levels of dopamine were seen in the treatment group. However, more clinical data is required to ensure its application in humans [71]. A similar study by Barcia et al. presented optimized ropinirole-loaded PLGA nanoparticles to treat the rotenone-induced Wistar mice model [168]. The nanoprecipitation was utilized for the synthesis of particles followed by morphology, surface charge, and loading characterization. The results from the evaluation suggested that the size of NPs was 152.2 nm, PDI of 0.29, and encapsulation of 74.8%. The surface charge of the particles was -14.25 mV and the amorphous state with confirmed by X-ray diffraction. The cumulative release from the optimized formulation was 20% in the first 24 h followed by controlled release for 5 days. The effectiveness of the formulation was tested, and the results showed no mortality with drug NPs, an improvement on the catalepsy and akinesia tests in treatment groups. Furthermore, the histopathological studies revealed an enhanced neuroprotective effect. Also, drug NPs depicted a significant increase in drug activity in oxidative stress, apoptosis, and alpha-synuclein aggregation assays [168].

Additionally, an in-situ gel-based long-acting injectable was developed by Zhao et al. for neuroprotective and slow PD progression. This PLGA-based gel system was optimized and subjected to in-vitro release and animal pharmacokinetic studies. The results from the in-vivo release in SD rats showed a Cmax of 64 ng/ml with a steady release for 30 days. Furthermore, the final formulation can be optimally administered using a 20 gauge (G) needle, demonstrating a viscosity of 2159 cp. This rasagiline-loaded sustain-release gel formulation can have a potential therapeutic advancement however more clinical data is needed to unravel its full potential [169]. Working on a similar line, Tunesi et al. investigated the collagen-hyaluronic acid-gelatin-based gel system for the delivery of Hsp70 in the management of PD. The gel characterization produced from the double desolvation method revealed an average size of 157.2 nm with high biocompatibility in SH-SY5Y cells from day 1st to day 7th. Cumulative release from the gel implant was around 95% after 96 h following the Korsmeyer-Peppas model. Moreover, the in-vivo inflammatory response studies in the brain revealed an increase in inflammation till 3rd day which was reduced on 7th day. However, the Tat-Hsp70 composites were diffused more rapidly with a greater target area than free protein. The data from the PD mouse model depicted a positive neuroprotective response in the treatment group. In conclusion, the study's outcome suggested positive therapeutic outcomes, but more extensive studies are required to understand the full potential of the project [170].

Xue et al. investigated the role of gelatin-polyaniline (PANI) hydrogel composites containing bone marrow stromal cells (BMSCs) to improve PD condition. The delivery composites were prepared by covalent and hydrogen bonding. The gel was subjected to gel degradation studies, in-vitro MTT assay and CCK-8 assay, and in-vivo animal treatment followed by behavioral studies like open field activity and rotarod test. The observations from the MTT and CCK-8 assays revealed that BMSCs-loaded gel was biocompatible. These hydrogel implants showed a protective effect in the MPTP-PD model, reducing dopaminergic neuron loss. Also, the BMSC gel illustrated a significantly higher release of GDNF in SNc after 28 days of delivery. Furthermore, improvement in motor activity in behavioral studies was observed indicating the potential of BMSC-hydrogel to treat PD. However, further extensive data is required to predict its efficacy in humans [171].

Mogharbel and co-workers interestingly studied the co-delivery of levodopa and curcumin for the treatment of Parkinson’s, where they prepared Poly(ethylene oxide)-poly(ε-caprolactone) (PEO-PCL) nanoparticle suspensions. The average particle size of NPs was 99.5 nm, the zeta potential was 25.6 mV, and the polydispersity index was 0.30 illustrating a stable delivery system. Furthermore, the encapsulation efficiency for both drugs curcumin and levodopa were 19.5% and 3.1% respectively. The total drug loading for curcumin was higher (97.7%) than the levodopa (10.4%) with no size change after loading. In addition to this, the hemolysis and MTT assays exhibited good biocompatibility and cell viability was above 95% in all concentrations at all time points. In conclusion, brain codelivery using co-block polymer showed promising results and can be used for better brain delivery in neurodegenerative diseases [172].

## Conclusion

This article provides an overview of possible technologies that can be used for delivering hydrophilic small and large molecules. Before deciding the long-acting technology complete understanding of drug molecules and characteristics of the delivery system is of prime importance. The key parameters are the properties of the drug molecules, their solubility, log P, pKa, and pharmacokinetic parameters, whereas factors dependent on the delivery system are whether local or systemic effect is required. For Systemic effect SC or IM route is feasible but for local effect, the formulation is directly administered at the site of action. In the case of the localised delivery duration of effect, size and the physical form of the dosage form needs to be compatible with the application site. The literature review of the approved long-acting injectables revealed that most of the drugs formulated as long-acting are potent with good physical and chemical stability in addition to broad therapeutic window and low water solubility. When it comes to designing hydrophilic molecules as long-acting it is very challenging compared to low-solubility drugs. Specially in this era where proteins have been used as therapeutic candidates possess a hydrophilic nature in addition to this they also show structural complexity and stability issues. Hence delivery of hydrophilic molecules using hydrophobic polymers comes with lots of challenges. The use of the emulsion technique can cause the degradation of labile proteins due to low pH use for maintaining the solubility or due to the organic solvent used in the preparation.

Novel techniques such as in-situ forming implants or solution-based formulations have made it easier to deliver labile molecules with easy manufacturing procedures. However, this system has been known to show higher variability in the in-vitro release profile and the pharmacokinetics of the drug. It could be due to the variation in the shape of the deport formed on administration which may lead to the clinical complexity requiring an increase in the size of the study group. To reduce the chances of failure and the duration of formulation development it is very necessary to anticipate the issues related to the manufacturing and the in-vitro and in-vivo performance of the drug product. Hence the establishment of the IVIVC which is the predictive mathematical model that helps in determining the relationship between the in-vitro drug release and the in-vivo performance of the drug product. The in-vitro release profile of the drug product may vary depending on the apparatus used, media, temperature, and sample loading method. Therefore selection of appropriate methods for release testing is of major importance while establishing IVIVC.

## Data Availability

Not applicable.
