# Correction: Long-acting parenteral formulations of hydrophilic drugs, proteins, and peptide therapeutics: mechanisms, challenges, and therapeutic benefits with a focus on technologies

**DOI:** 10.1007/s13346-024-01781-w

**Published:** 2024-12-28

**Authors:** Deepa D. Nakmode, Baljinder Singh, Sadikalmahdi Abdella, Yunmei Song, Sanjay Garg

**Affiliations:** Centre for Pharmaceutical Innovation, University of South Australia, North Terrace, Adelaide, SA 5000 Australia

**Drug Delivery and Translational Research**

10.1007/s13346-024-01747-y

The article “Long-acting parenteral formulations of hydrophilic drugs, proteins, and peptide therapeutics: mechanisms, challenges, and therapeutic benefits with a focus on technologies“, written by Nakmode et al., was originally published electronically on the publisher’s internet portal on December 11, 2024, without open access. With the authors’ decision to opt for Open Choice the copyright of the article changed on December 18, 2024 to © The Author(s) 2024 and this article is licensed under a Creative Commons Attribution 4.0 International License, which permits use, sharing, adaptation, distribution and reproduction in any medium or format, as long as you give appropriate credit to the original author(s) and the source, provide a link to the Creative Commons licence, and indicate if changes were made. The images or other third party material in this article are included in the article's Creative Commons licence, unless indicated otherwise in a credit line to the material. If material is not included in the article's Creative Commons licence and your intended use is not permitted by statutory regulation or exceeds the permitted use, you will need to obtain permission directly from the copyright holder. To view a copy of this licence, visit http://creativecommons.org/licenses/by/4.0/.

